# The change in children's subjective relational social cohesion with family and friends during the COVID-19 pandemic: A multinational analysis

**DOI:** 10.3389/fsoc.2022.974543

**Published:** 2022-12-02

**Authors:** Oliver Nahkur, Dagmar Kutsar

**Affiliations:** Institute of Social Studies, Faculty of Social Sciences, University of Tartu, Tartu, Estonia

**Keywords:** relational social cohesion, social distancing, COVID-19 pandemic, quantity of relationships, quality of relationships, change profile

## Abstract

As a response to the COVID-19 pandemic, social-distancing measures have been implemented worldwide, including school closures. Previous studies indicated that children's relational social cohesion with family (RSC-Fa) and friends (RSC-Fr) may have decreased during the pandemic, but some children described that positive experiences were gained from the confinement measures of social distancing. Mostly, these studies are qualitative or capture a single country and have an exploratory character. Using data collected in 2021 of more than 20,000 children primarily aged 9–13 years as part of the International Children's Worlds COVID-19 Supplement Survey from 18 countries (Germany, Turkey, Bangladesh, Italy, Albania, Romania, Chile, Wales, Taiwan, Belgium, Algeria, Israel, Russia, South Korea, Indonesia, Estonia, Finland, and Spain), this study aimed to examine how the COVID-19 pandemic has affected children's RSC-Fa and RSC-Fr and explore the role of relational factors. RSC-Fa and RSC-Fr are measured through satisfaction in relationships with family members and friends before and during the COVID-19 pandemic, respectively. We employed descriptive statistics, cluster analysis, and multinomial logistic regression analysis. Our analyses confirmed the decrease in RSC-Fa and RSC-Fr, with a noticeably bigger decrease in RSC-Fr. Five profiles of change in RSC emerged: (1) gainers in both RSC; (2) gainers in RSC-Fa and decliners in RSC-Fr; (3) no change in either RSC; (4) decliners in RSC-Fa and gainers in RSC-Fr; and (5) decliners in both RSC. The quantity and quality of children's relationships differ by their profiles of change in RSC. For example, it was significantly more likely that “decliners in both RSC” had to be at home all day because of COVID-19 than “gainers in both RSC” or “no changers.” Mainly, the quantity of relationship factors, and among different quality factors, only autonomy perceptions, help to explain the children belonging to the “gainers in both RSC” profile compared to the “no changers.” Meanwhile, almost all the quantity and quality of relationships factors help to explain children's belonging to the “decliners in both RSC” profile compared to “no changers.” In conclusion, our study confirmed the importance of keeping schools open to protect the RSC of children.

## Introduction

Children are active agents who construct their own cultures and contribute to the production of the adult world (Corsaro, 2011). As agents, they “do things” with other people (Mayall, 2002), being self-determined and autonomous (Frønes, 2016). Thus, children need to be socially related (inter)generationally to be socially coherent. However, as a response to the COVID-19 pandemic, most countries in the world implemented social-distancing measures and ordered the lockdown of all residents, including school closures affecting more than 500 million students worldwide (Agarwal and Sunitha, 2020), to slow the rate of transmission, ease the pressure on the healthcare system, and protect at-risk populations (Armitage and Nellums, 2020). In some countries, children could leave home for sports or walks with their parents or guardians, while in other countries, these activities were prohibited (Garcia, 2020). For example, in the spring of 2020, Spain was the only European country where children were not allowed to leave their homes (Granda, 2020; Grechyna, 2020). The social-distancing measures affected children's social contact and changed their relational patterns, putting relational social cohesion—the quality and quantity of relationships—to test both inside and outside of the family group. Disconnection from social contacts curbs social development, including the social competencies of children. This may cause the deterioration of mental health revealed in many studies on pandemic outcomes (Fegert et al., 2020; Chaabane et al., 2021; Gadermann et al., 2021; O'Sullivan et al., 2021). According to a review by Loades et al. (2020), the pandemic increased children's mental health problems, especially related to loneliness and social isolation, a conclusion that highlights the importance of protecting relational social cohesion during periods of social distancing.

Social-distancing measures, including school closures, may have had different effects on children's relational social cohesion (inter)generationally, i.e., with family and friends. Measures limited in-person contact with friends and extended family while increasing it with immediate family (Chaabane et al., 2021; Kutsar and Kurvet-Käosaar, 2021; Shah et al., 2021). Online tools have been increasingly used to compensate for the lack of in-person interactions with friends and extended family. However, there is some evidence from South Korea (Choi et al., 2021) and Switzerland (Stoecklin et al., 2021) that the quality of relationships with friends decreased as an outcome of the confinement measures, while school and workplace closures meant that family members spent more time together in greater proximity, resulting in shared social isolation, anxiety, stress, and conflict (Biroli et al., 2020; Lebow, 2020). Still, sharing new circumstances could also lead to increased closeness between family members, especially in cases of high pre-pandemic intra-familial closeness (Mariani et al., 2020) or due to new shared activities (Salin et al., 2020; Kutsar and Kurvet-Käosaar, 2021; Stoecklin et al., 2021).

Previously, the effect of the COVID-19 pandemic on children's relationships with their friends and family has been explored in a single country, e.g., in South Korea (Choi et al., 2021), Germany (Vogel et al., 2021), Finland (Salin et al., 2020), Spain (Mondragon et al., 2021), and Estonia (Kutsar and Kurvet-Käosaar, 2021). There are also some multinational qualitative studies (e.g., Shah et al., 2021; Stoecklin et al., 2021). Stoecklin et al. (2021) examined children's experience of the lockdown in relation to their family life and contacts with friends in Switzerland, Canada, and Estonia. Shah et al. (2021), in their longitudinal ethnographic action research, focused on children aged 14–18 years and how their agency shaped family dynamics during the COVID-19 pandemic in Italy, Lebanon, Singapore, and the United Kingdom. However, there is no evidence that the decrease in children's relational social cohesion during the COVID-19 pandemic is a common feature across countries.

Thus, it is likely that, among children, different profiles of change in relational social cohesion with family and friends emerged, e.g., for some children, their relational social cohesion with friends decreased, while with their family, it increased, but for some other children, the decrease was evident with both family and friends. In the present study, we focused on relational social cohesion and used data collected in 2021 from more than 20,000 children primarily aged 9–13 years from 18 countries across the globe as the part of International Children's Worlds COVID-19 Supplement Survey. The aim was to examine how the COVID-19 pandemic has affected children's relational social cohesion with family and friends from their perspectives. To our knowledge, this is the first such quantitative study based on such a large-scale and multinational sample.

In this study, we first provide an overview of the construct of social cohesion and previous evidence on children's relational social cohesion with friends and family during the COVID-19 pandemic. We conclude this by describing gaps in previous studies and introducing our research questions. Second, we describe the sample and measures used for the International Children's Worlds COVID-19 Supplement Survey and the methods of data analysis. Third, we present the findings to answer the research questions. The study ends with a discussion and conclusions.

## Social cohesion

Social cohesion is “a multidimensional construct consisting of phenomena on the micro (e.g., individual attitudes and orientations), meso (features of communities and groups), and macro (features of societal institutions) level” (Schiefer and van der Noll, 2017, p. 583). According to the review by Schiefer and van der Noll (2017), six dimensions of social cohesion are most common: social relations, identification, orientation toward the common good, shared values, quality of life, and (in)equality. However, according to Dragolov et al. (2016) and Schiefer and van der Noll (2017), the essential dimensions of social cohesion are the first three: (1) the quality of social relations, (2) identification or connectedness with the social entity, and (3) orientation toward the common good. In this study, we focused on the most prominent dimension of social cohesion (Schiefer and van der Noll, 2017)—social relations, also called relational social cohesion (Moody and White, 2003; Janmaat, 2011), on the micro level, encompassing relationships between individuals.

Both Dragolov et al. (2016) and Schiefer and van der Noll (2017) consider social networks, trust in other people, and acceptance of diversity as important components of social relations. We are particularly interested in social networks—the quality and quantity of children's relationships with their family and friends. According to the Australian Bureau of Statistics (2006, p. 19), especially “[…] the quality and strength of people's relationships and bonds with others—their family, friends, and the wider community—are important ingredients of the level of social cohesion.” Thus, in a cohesive society, children have high-quality relationships with their friends and family, as well as a sufficient quantity of them.

Social networks are important in children's lives. Children are, on the one hand, embedded in the social networks of their families and, on the other hand, create their own networks in which they spontaneously participate. According to Corsaro (1997), the “individual development of children is embedded in the collective production of a series of peer cultures which in turn contribute to reproduction and change in the wider adult society or culture” (p. 26). The latter means that children, besides their family of origin, participate in other institutional locales with other people (children and adults) who are not their family members. As Corsaro (1997) characterizes it, children “weave their webs” (p. 24). We argue that social-distancing measures during the pandemic affected these processes. More specifically, with reference to Dragolov et al. (2016) and Schiefer and van der Noll (2017), we contend that social-distancing measures reshaped the social networks of children and, thus, affected levels of social cohesion.

Besides in-person networking, children participated in internet social networks, which have become an important component of children's subculture (see, e.g., Stasova and Khynova, 2012). Does internet social networking limit the influence of physical social isolation during the pandemic and help social coherence?

All of the above creates the impression of a normative approach: every child is actively embedded in social networks (intra-familial and beyond; in-person and virtual). Being connected gives children a sense of belonging and trust in other people and develops their social and other skills. The meaning of a child who is actively embedded in different networks, i.e., is socially coherent definitely has a positive social connotation. However, not all children have good relationships with family members and not all children are actively embedded in external social networks. Moreover, some children are “self-omitters” from peer relationships (Hall et al., 2021). The latter was more often classified as being bullied in a study by Hall et al. (2021), and, at least in the classroom, their social cohesion cannot be high. In addition, studies about inclusive schools have demonstrated the low relational social cohesion of children with special needs (e.g., Locke et al., 2010; Kasari et al., 2011). Thus, there are grounds to suppose that not all children can meet the “standards of normalcy” of being socially active and highly relationally socially coherent, as adults put it. We argue that formal social isolation could be a method of escape for these children, and they could probably, subjectively, gain from the pandemic. However, this does not mean that they would gain a sense of belonging, trust other people, or develop communication skills. Those neglected by their peers or the “self-omitters,” thus, could gain even more from social distancing when living with family members who are friendly and understanding. However, in the context of bad family relationships, such as children experiencing neglect or abuse, their status as “self-omitters” is evident and may even solidify during formal social isolation in their home.

## Previous evidence on children's relational social cohesion with family and friends during the COVID-19 pandemic

Children interact in different life domains, including family, school, and friendship groups, in-person or through technology. The COVID-19 pandemic caused momentous changes in patterns of interaction in children's lives due to the implementation of lockdowns and policies on social distancing. There is some, primarily qualitative, evidence that children's relational social cohesion with friends and family changed during the pandemic. For example, Stoecklin et al. (2021) examined children's experience of lockdown in relation to their family life and contacts with friends in Switzerland, Canada, and Estonia. They found that lockdown influenced children's quality of relationships with their friends and family, but to a different extent. For example, in Switzerland, half of the respondents said that their social life with friends stayed more or less the same, while 79% of the respondents declared no change in their family life. Shah et al. (2021) demonstrated that, in different countries, young people living in families with close and stable relationships found it easier to cope with the pandemic circumstances; by contrast, living in close proximity exacerbated family tensions and conflicts and endangered intra-familial closeness.

Next, we describe previous evidence on changes in children's quantity and quality of relationships in families and with friends during the pandemic and outline gaps in the research.

### Change in the quantity and quality of relationships in families

In the context of children's relationships with their family members, lockdown restrictions functioned mainly as drivers of physical density in their homes. The fear of getting infected or infecting others, “COVID-19 anxiety,” may have amplified the social isolation of the whole family. According to children's perceptions, interaction with family members has increased in quantity. For example, in Estonia, in spring 2021 compared to spring 2020, children more often complained about having to spend time with their family members 24/7, resulting in tense family relationships and arguments and occasional conflicts with younger siblings (Kutsar and Kurvet-Käosaar, 2021). Thus, by spring 2021, the physical density in the homes had worsened the atmosphere within the families.

However, social-distancing regulations have also affected the quality of children's relationships with their family members differently (Kutsar and Kurvet-Käosaar, 2021). Some children experienced more and better time with family members; parents were seen as an important source of support during the lockdown period, and many said that this period brought them closer to their parents (Salin et al., 2020), especially during the first lockdown in spring 2020 (Stoecklin et al., 2021).

For some children, disputes and conflicts with other family members became more frequent. For example, South Korean schoolchildren reported experiencing more conflicts, worries, and scolding from their parents during the pandemic (Lee et al., 2020). In Australia, about a quarter of the adolescents surveyed reported that conflicts with their parents had increased during the lockdown period and half of the sample reported an increase in conflicts with their siblings (Magson et al., 2021). In Estonia, about a third of children reported an increase in anxiety and tension in relationships at home (Kutsar and Kurvet-Käosaar, 2021). According to Stoecklin et al. (2021), the sources of these tensions were that children felt they lacked their own space and privacy and/or experienced more intense parental control as an impediment to their autonomy.

Some children reported being left alone or being lonely, e.g., stemming from many meaningful relationships that were put on hold during the lockdown period, for example, with extended families, such as grandparents (Stoecklin et al., 2021). Missing their extended family was more frequent among younger children (Kirsch et al., 2020). In Estonia, children were most often concerned about the lives of their grandparents, who the children understood belonged to the group at-risk of fatal outcomes from contracting the virus and whom they could not visit (Stoecklin et al., 2021). In sum, the pandemic endangered children's familial relational social cohesion.

### Change in the quantity and quality of relationships with friends

Keeping in-person distance from friends was the most difficult challenge during the pandemic and lockdown according to children (Ellis et al., 2020; Kutsar and Kurvet-Käosaar, 2021; Magson et al., 2021; Stoecklin et al., 2021). Confinement measures of social distancing decreased the quantity of children's in-person interactions with their friends because of temporarily losing physical access to schools, playgrounds, and recreational activities (Stoecklin et al., 2021). Thus, in the context of children's relationships with their friends, the policies of social distancing functioned as drivers of compulsory physical separation. The severity of measures differed from country to country. For example, in Spain, all children experienced extreme lockdown for up to 5 weeks in the spring of 2020 (Garcia, 2020; Granda, 2020; Grechyna, 2020), as they were forbidden from leaving their homes. Less extreme and more common was the requirement to stay at home when a child or his/her close contact (e.g., a family member, or classmate) was infected with COVID-19.

Despite the existence or non-existence of drivers of compulsory physical separation, children may have self-chosen to limit in-person contact with their friends, e.g., because of the “COVID-19 anxiety,” such as the fear of being infected or infecting others. For example, in Germany, younger children were more afraid of COVID-19 and worried more about themselves, family, and friends than older children, and girls were more afraid of COVID-19 and more worried about their friends than boys (Vogel et al., 2021). However, most children and adolescents worried more about their families rather than themselves (Vogel et al., 2021). With the heightened virus risk perception, children may not feel safe during in-person interactions with friends and, thus, prefer to maintain physical distance. We consider these factors as drivers of physical self-distancing or becoming “self-omitters” (a term defined by Hall et al., 2021).

Although the quantity of children's virtual interactions with friends using smartphones (Munasinghe et al., 2020; Sañudo et al., 2020) and social media (Ellis et al., 2020) increased during the pandemic to compensate for physical distancing, for many children, virtual communication with friends could not substitute regular in-person contact (Kutsar and Kurvet-Käosaar, 2021; Stoecklin et al., 2021). However, the lockdown also led to the creation of new individual friendships, evident in “COVID-19 relationships,” e.g., those formed between two to three families in the neighboring area and their children (Stoecklin et al., 2021).

There is some evidence that the quality of relationships with friends decreased during the COVID-19 pandemic. For example, this phenomenon is documented in South Korea (Choi et al., 2021) and in Switzerland (Stoecklin et al., 2021), where four out of ten children stated that their social life with friends was getting worse. Family isolation and social distancing were felt to be the cause of the decline in the quality of friendships (Stoecklin et al., 2021). There is some evidence of other possible causes for the decline in the quality of friendships. According to Vogel et al. (2021), during the pandemic, the perceived social support from peers decreased shortly after the lockdown, and it was more pronounced for younger children and those from a medium/low socio-economic background. Older children have more availability of electronic devices and social platforms (Auhuber et al., 2019), and older children may be less compliant with social-distancing guidelines (Goldstein and Lipsitch, 2020). Thus, especially for older children, feeling unsafe during in-person interactions with their friends may be also important.

According to Kutsar and Kurvet-Käosaar (2021), by spring 2021, the quality of relationships with friends had clearly worsened. Some children explained that they do not know what to say to their friends, as they no longer share their daily lives, do not really know how to keep in touch, and miss playing in a group (Stoecklin et al., 2021). Many children felt estranged from their friends (Kutsar and Kurvet-Käosaar, 2021), although still missing them (Kutsar and Kurvet-Käosaar, 2021; Stoecklin et al., 2021; Larivière-Bastien et al., 2022). For example, in Germany, about 80% of children missed in-person contact with friends (Vogel et al., 2021). Missing their friends was more frequent among older children (Kirsch et al., 2020) and was described as a strong feeling (Stoecklin et al., 2021). Especially challenging were separations from their boyfriend or girlfriend due to confinement measures (Kutsar and Kurvet-Käosaar, 2021; Stoecklin et al., 2021). Missing friends or classmates caused children to experience feelings of loneliness (Jiao et al., 2020; Okruszek et al., 2020; Singh and Singh, 2020), and even online school did not satisfy the same needs for daily social interactions (Larivière-Bastien et al., 2022). Loneliness is an exceedingly painful experience that is the result of an unfulfilled need for closeness and social relationships that are felt to be insufficient or not entirely satisfactory (Berger and Poirie, 1995). Therefore, the emergence of this feeling indicates that, in children, disconnection from in-person contact with friends and classmates makes them feel lonely: they miss the opportunity for such interaction, or, at least, they do not have sufficient opportunities. Some children said that they had lost all their friends and were now completely alone (Kutsar and Kurvet-Käosaar, 2021), i.e., their relational social cohesion with friends had suffered. For example, in Germany, the percentage of children who had no contact with their peers (in-person or online) increased from 3% pre-COVID-19 to 14% in April, 2020 (Vogel et al., 2021).

### Gaps in previous evidence and research questions

Previously, the effect of the COVID-19 pandemic on children's relationships with their friends and family has been explored in single-country studies (Salin et al., 2020; Choi et al., 2021; Kutsar and Kurvet-Käosaar, 2021; Mondragon et al., 2021; Vogel et al., 2021). There are also some multinational qualitative studies (e.g., Shah et al., 2021; Stoecklin et al., 2021). However, to our knowledge, there is no evidence of how the change in children's relational social cohesion with family and friends during the COVID-19 pandemic has varied between countries. Thus, our first research question is:

- RQ1: How has children's relational social cohesion with family and friends changed during the COVID-19 pandemic?

Inspired by the previous research evidence described in sections “Change in the quantity and quality of relationships in families” and “Change in the quantity and quality of relationships with friends,” we hypothesize that children's relational social cohesion decreased more with friends than within family.

Previous studies indicated that children's relational social cohesion with friends (e.g., Choi et al., 2021; Stoecklin et al., 2021) and in families (e.g., Kutsar and Kurvet-Käosaar, 2021) may have decreased during the COVID-19 pandemic, but some children still described positive experiences gained from the confinement measures of social distancing (Salin et al., 2020). Thus, some children gained from the pandemic in terms of the quantity and quality of relationships in the family, but lost friends; some lost both in families and with friends. There is also some evidence that children's quality of relationships with their friends and family did not change much (Stoecklin et al., 2021). However, there seems to be no evidence of whether some children gained from the pandemic in terms of the quantity and quality of relationships with family and with friends or gained with friends and lost in the family. Moreover, we are not aware of any previous study determining different profiles of changes in children's subjective relational social cohesion with family and friends experienced during the COVID-19 pandemic. Thus, our second research question is:

- RQ2: What profiles of change in children's relational social cohesion have emerged during the pandemic?

We claim that it requires a “large *N*” sample to obtain an overview of all the possible profiles of change and consider our country-pooled sample (*N* > 20,000) suitable for that kind of analysis. Country differences in profiles of change are not considered in this study due to high variation in sample sizes and small *N* values in some countries.

Exploring the quantity and quality of relationships by profiles of change, including what relational factors help to explain children's belonging to a certain profile of change in relational social cohesion, offers a new insight to better support children in such exceptional times. Our third and fourth research questions are:

- RQ3: How do the profiles of change in relational social cohesion differ by children's quantity and quality of relationships in the family and with friends?
- RQ4: What relational factors can help to explain children's belonging to a certain relational social cohesion profile?

Regarding research questions 2–4, we adopted a more exploratory approach in examining “profiles of change” without establishing extra hypotheses.

## Data and methods

### Data source and sample

The study gathered data from the International Children's Worlds COVID-19 Supplement Survey collected in 2021, primarily from children aged 9–13 years. The first version of the database included children's data from the following 20 countries: Germany, Turkey, Bangladesh, Italy, Albania, South Africa, Romania, Chile, Wales, Colombia, Taiwan, Belgium, Algeria, Israel, Russia, South Korea, Indonesia, Estonia, Finland, and Spain. We excluded South Africa and Colombia due to the absence of data on some measures that we considered important for our analyses. The final sample consisted of data from over 20,000 children from 18 countries (Table 1). The period of data collection varied slightly between countries (Table 1), but mostly it was collected between the peaks of the second and third waves. Turkey was one of the countries where children reported most often that there were times when they had to be in their homes all day because of COVID-19, and they could not attend school for many days. It was the opposite in Finland. Data-collection methods varied from country to country between pencil and/or web survey methods. Due to the difficulties in collecting data from children during the COVID-19 pandemic (and during the (semi) lockdown in many countries), representative samples were mostly not achieved. Different sampling methods were used, i.e., stratified (in Belgium) or cluster (in South Korea) as a representative, and convenience (e.g., in Taiwan, Bangladesh, Indonesia, Israel), purposive (in Chile), and snowball (in Germany) as non-representative, sampling methods. In some cases, only country regions were captured. In addition, sample sizes vary broadly from 590 in Germany to 2,422 in Belgium.

**Table 1:** Countries' sample representativeness, geographical coverage, data collection method (PPS-paper-pencil survey; WS-web survey), total number of children, including proportions (%) by gender, frequency of access to the Internet, not having own room, and experiences of social-distancing measures.

	**Representative sample—yes or no**	**Geographical area covered by sampling strategy**	**Data collection time in 2021**		**Data collection method**		**Total number of children**		**Gender**			**Access to the internet during COVID-19**	**Having own room**	**There were times where I had to be in my home all day because of COVID-19**	**I could not attend school for many days**
			**Start**	**End**	**PPS (in person) %**	**WS (PC/tablet /mobile phone) %**	***N***	**%**	**Boys, %**	**Girls, %**	**Binary, %**	**Often always %**	**No, %**	**Yes, %**	**Yes, %**
Albania	No	The capital of Albania, Tirana in urban and rural areas	22.06	30.07	73.2	26.8	1,034	4.7	54.5	45.5	0	82.0	29.1	76.9	84.2
Algeria	Yes	Province of Oran	2.11	16.12	100		816	3.7	52.3	47.7	0	51.4	59.2	63.5	67.3
Bangladesh	No	Mainly regions of Barishal, Moulvibazar, Rajshahi and Dhaka (capital)	10.08	31.08	78.0	22.0	1,370	6.3	50.4	49.6	0	35.3	49.6	68.7	91.6
Belgium	Yes	Whole Flemish community in Belgium (Flemish region and the Dutch speaking population in Brussels)	25.05	29.06		100	2,422	11.1	50.6	49.4	0	89.3	14.8	78.0	81.2
Chile	No	Metropolitan region of the cities of Santiago and Concepción (also Curicó, Quilpué and, Laja cities)	30.08	8.10	4.4	95.6	1,682	7.7	47.8	49.2	3.1	91.1	20.5	75.4	87.1
Estonia	No	Whole country	21.04	7.06		100	1,258	5.8	50.0	47.8	2.2	97.7	17.7	66.1	25.2
Finland	No	Southwestern Finland (Turku and Naantali)	19.04	2.06		100	1,003	4.6	47.9	51.0	1.1	93.2	17.5	34.3	29.4
Germany	No	Whole country with a focus on Frankfurt/Hessen	25.10	29.11		100	590	2.7	51.2	48.1	0.7	87.1	7.8	48.4	96.9
Indonesia	No	West Java Province	17.07	14.09		100	2,222	10.2	53.9	46.1	0	48.5	37.6	61.9	88.5
Israel	No	Whole country	Wave1: 30.05 Wave2: 30.09	Wave1: 27.06 Wave2: 20.10	100		930	4.3	47.0	50.7	2.3	87.1	32.8	72.2	76.7
Italy	No	Whole country but mainly the cities of Genoa and Rome and southern regions of Campania, Calabria, and Puglia.	End of May	30.09		100	919	4.2	49.6	50.4	0	95.7	35.4	58.2	98.4
Romania	Yes (mix between convenience and representative sample)	Whole country	20.05	15.06	100		1,856	8.5	51.2	48.8	0	92.0	40.3	66.8	76.8
Russia	Yes	Tyumen region	10.05	25.05		100	876	4.0	50.5	49.5	0	93.7	17.5	75.6	76.8
S Korea	Yes	Whole country	22.07	20.08		100	1,497	6.9	48.9	51.1	0	91.6	5.8	58.8	26.5
Spain	No	Province of Girona	5.05	4.08	59.8	40.2	702	3.2	49.3	48.3	2.3	87.6	21.0	76.9	86.2
Taiwan	No	Whole country	26.07	10.09		100	1,155	5.3	54.4	45.5	0.2	81.4	47.9	81.0	29.8
Turkey			8.06	30.08	50.5	49.5	804	3.7	49.8	49.2	1.0	87.3	32.2	85.9	93.6
Wales	No	Rural North, Rural Heartland, Metropolitan Wales, and Valleys	5.07	15.07		100	691	3.2	45.7	50.8	3.5	96.6	11.7	79.4	78.2
Total							21,827	100	50.5	48.7	0.8	81.0	28.0	68.3	71.5

### Measures

We measured the relational social cohesion at the micro level in families (RSC-Fa) and with friends (RSC-Fr) before the COVID-19 pandemic with children's subjective retrospective assessments—“Satisfaction before COVID-19 with the relationships I had with people I live with” and “Satisfaction before COVID-19 with the relationships I had with my friends”—and during the pandemic with “Satisfaction now during COVID-19 with the relationships I have with my friends” and “Satisfaction now during COVID-19 with the relationships I have with people I live with.” An 11-point assessment scale was used, where 0 was “not at all satisfied” and 10 “totally satisfied.” Changes in RSC-Fa and RSC-Fr for each child were computed as follows: “RSC now”—“RSC before the COVID-19.”

In Table 2, the quantity and quality of relationship factors used as independent variables are described. In the case of all items, lower values refer to a lower quantity and quality of relationships. Some items on quantity (e.g., the experience of quarantine) and quality (e.g., having problems with siblings, missing friends, classmates, and relatives) were not used due to the absence of data in many countries.

**Table 2:** Quantity and quality of relationships factors used as possible predictors of RSC change (all measures low-> high).

**Factors**	**Items**	**Scale**
	**Quantity of relationships**	
	*Friends + family*	
Compulsory physical distancing from friends, and a high density of contacts inside the family due to the confinement measures	There were times where I had to be in my home all day (including the garden, yard, or balcony, if you have) because of the Coronavirus I could not attend school for many days	
In-person self-distancing from friends, and a high density in family due to infection or risk of infection	Me or somebody in my home got infected with Coronavirus At home, we had to be very careful because somebody was considered at high risk of getting very ill if they got infected with the Coronavirus	1-yes, 2-not sure, and 3-no
In-person self-distancing from friends, and a high density in family due to COVID-19 anxiety	I am very afraid of the Coronavirus It makes me uncomfortable to think about the Coronavirus My hands become sweaty when I think about the Coronavirus I am afraid of losing my life because of the Coronavirus When I watch news and stories about the Coronavirus on TV and social media, I become nervous or anxious I cannot sleep because I'm worrying about getting the Coronavirus My heart races (beats very fast) when I think about getting the Coronavirus	0-I totally agree, 1-I agree a lot, 2-I agree somewhat, 3-I agree a little, and 4-I do not agree Arithmetic mean of these items
	*Friends*	
Frequency of in-person or online interactions	Playing or hanging out outside During the Coronavirus how often spend time meeting with your friends online (e.g., on the computer, zoom, or any other way)	0-never, 1—less than once a week, 2—once or twice a week, 3—3 or 4 days a week, 4—5 or 6 days a week, and 5—every day
New online friendships	I made new friends with other children online during the Coronavirus	0—I do not agree, 1—I agree a little, 2—I agree somewhat, 3—I agree a lot, and 4—I totally agree
	**Quality of relationships**	
Perceptions of safety	I feel safe with my friends	0—I do not agree, 1—I agree a little, 2—I agree somewhat, 3—I agree a lot, and 4—I totally agree
	I feel safe at home	0—I totally agree, 1—I agree a lot, 2—I agree somewhat, 3—I agree a little, and 4—I do not agree
Perceptions of support	During the Coronavirus, I felt well-supported by some of my friends	0—extremely … 10—not at all
	During the Coronavirus, I felt well-supported by some people I live with	
Perceptions of loneliness	I feel alone	0—not at all satisfied … 10—totally satisfied
Perceptions of boredom	How much you have felt this way during the last 2 weeks – bored?	0—I do not agree, 1—I agree a little, 2—I agree somewhat, 3—I agree a lot, and 4—I totally agree
Perceptions of autonomy	Satisfaction with the freedom you have	
Perceptions of “being listened to'	My opinions about the Coronavirus are taken seriously in my home	

### Data analyses

In this paper, we processed data to address the research questions in four steps. As we did not expect that a child had values for all variables, *N* varies in each step of our analyses. Compared to other countries, Germany was the country where missing values were the most common problem.

First, to answer the first research question (“How have children's relational social cohesion with family and friends changed during the COVID-19 pandemic”), we examined the means and the percentages of low (“0–4”) and maximum (“10”) RSC-Fa and RSC-Fr values, by country and in total, (1) before and (2) during the COVID-19 pandemic. We considered values 9–10 as “very high,” 8 “high,” 7 “average” 6 “low,” and 5, “very low.” By subtracting the “before” from the “during the COVID-19 pandemic” value, each child was attributed a change in RSC-Fa and RSC-Fr values. Out of 21,827 children, for 711 and 707 we were not able to compute the change of RSC-Fa and RSC-Fr values, respectively, due to missing data. Countries were ranked based on their level of the average change in RSC-Fa and RSC-Fr. To answer the second research question (“What profiles of change in children's relational social cohesion have emerged during the pandemic?”), based on the average change in RSC-Fa and RSC-Fr during the pandemic, we conducted a cluster analysis using country-pooled data. We called the clusters “profiles of change in relational social cohesion.” We used a two-step cluster analysis in SPSS 28 with Euclidean distance and without a fixed number of clusters. It requires a “large *N*” sample to obtain an overview of all the possible profiles of change, and we considered our country-pooled sample (*N* > 20,000) suitable for this kind of analysis. For 856 children, the profile of change was not attained due to the missing data.

To answer the third research question (“How do the profiles of change in relational social cohesion differ by children's quantity and quality of relationships in the family and with friends?”), differences in children's quantity and quality of relationships in the family and with friends between the change profiles were assessed using the Kruskal–Wallis test, which is based on analyzing the mean rank. When a significant difference was found, *post-hoc* tests were conducted using Mann–Whitney's *U*-test to assess the differences between each pair of the profile. The difference was considered statistically significant when *p* < 0.05. We used nonparametric tests because our variables do not meet normal distribution criteria and the size of the profiles differ markedly. The missings varied by variable, from 1,112 (perception of home safety) to 1,922 (COVID-19 anxiety).

To answer the fourth research question (“What relational factors can help to explain children's belonging to a certain relational social cohesion profile?), multinomial logistic regression analysis was used. We used the children's gender (1 = girls and 2 = boys; non-binary children were excluded due to the small group size), frequency of access to the Internet, and existence of their own room as controls. The age of the children was not included as a control as we predominantly had data for 9–13 years-old children but only 8 children aged 7 or 8 and 155 children aged 14 or 15. Children's profiles of change in relational social cohesion were used as the dependent variable in the regression model. We included “gainers in both RSC” (102 missings), “no changers” (3,592), and “decliners in both RSC” (407) profiles. “No changers” was the reference group. The other two profiles were excluded due to the small *N* (< 100). As 11 countries out of 18 had fewer than 100 children in the “decliners in both RSC” profile (the second most populous behind “no changers'), we decided not to run regression models for each individual country.

## Results

### The change in children's subjective relational social cohesion with family during the COVID-19 pandemic

In total, children's subjective relational social cohesion with family members (RSC-Fa) did not change much, as it decreased by only 0.5 points on the 11-point scale, remaining at a high level (Table 3). It decreased by more than 1 point on the 0–10 scale only in Turkey and Bangladesh, where 16 and 18% of children assessed their RSC-Fa as low during the pandemic, respectively. In Spain, RSC-Fa did not decrease at all.

**Table 3:** Means and % of low and high relational social cohesion with family members (RSC-Fa) by country and in total before and during the COVID-19 pandemic, and children's RSC-Fa mean change (countries listed by the change in RSC-Fa in decreasing order).

	**RSC-Fa before pandemic (*****N*** = **21,449)**			**RSC-Fa during pandemic (*****N*** = **21,389)**			**Change in RSC-Fa (*N* = 21,116)**
	***M* (SD)**	**Low (“0–4”) RSC-Fa, %**	**Highest (“10”) RSC-Fa, %**	***M* (SD)**	**Low (“0–4”) RSC-Fa, %**	**Highest (“10”) RSC-Fa, %**	***M* (SD)**
Turkey	8.76 (1.9)	4.2	53.2	7.42 (2.9)	15.7	37.1	−1.35 (2.8)
Bangladesh	8.46 (2.6)	9.4	57.4	7.45 (3.0)	17.7	40.9	−1.01 (2.9)
Germany	8.92 (1.7)	3.0	51.3	7.83 (2.4)	10.9	33.8	−0.90 (2.9)
Albania	9.47 (1.3)	1.0	76.9	8.67 (2.2)	5.8	55.3	−0.80 (2.3)
Italy	9.19 (1.6)	2.8	65.4	8.56 (2.2)	5.7	52.2	−0.63 (2.0)
Chile	8.8 (2.2)	5.8	62.5	8.25 (2.6)	10.2	52.6	−0.56 (2.2)
Wales	8.63 (2.2)	7.3	57.1	8.07 (2.6)	11.6	45.2	−0.55 (2.3)
Taiwan	8.39 (1.9)	3.6	41.0	7.92 (2.6)	8.8	38.6	−0.47 (2.1)
S Korea	7.53 (1.4)	1.9	5.7	7.08 (1.7)	7.4	4.6	−0.45 (1.5)
Belgium	8.5 (2.4)	7.7	54.4	8.11 (2.6)	10.7	46.7	−0.40 (2.1)
Indonesia	8.86 (2.1)	5.4	60.2	8.47 (2.4)	8.4	53.0	−0.39 (2.0)
Romania	9.24 (1.8)	3.8	75.4	8.91 (2.2)	5.4	65.5	−0.37 (2.0)
Algeria	7.91 (3.2)	14.9	54.7	7.57 (3.3)	18.0	48.8	−0.34 (3.5)
Estonia	8.81 (1.9)	4.6	53.9	8.48 (2.2)	7.4	49.3	−0.34 (1.6)
Russia	8.08 (2.8)	13.7	51.6	7.77 (3.0)	16.8	47.3	−0.30 (1.8)
Finland	9.1 (1.7)	2.7	62.1	8.87 (1.9)	4.2	55.8	−0.24 (1.3)
Israel	8.62 (2.4)	8.2	60.7	8.52 (2.5)	8.9	58.5	−0.11 (2.4)
Spain	8.28 (2.5)	10.8	50.0	8.44 (2.4)	7.7	53.8	0.21 (2.5)
Total	8.65 (2.2)	5.9	55.4	8.16 (2.5)	9.8	46.9	−0.49 (2.2)

### The change in children's subjective relational social cohesion with friends during the COVID-19 pandemic

In total, children's subjective relational social cohesion with friends (RSC-Fr) was at a high level before the pandemic but it decreased by 1.3 points on the 11-point scale, to be between the low and average levels during the pandemic (Table 4).

**Table 4:** Means and % of low and high relational social cohesion with friends (RSC-Fr) by country and in total before and during the COVID-19 pandemic, and children's RSC-Fr mean change (countries listed by the change in RSC-Fr in decreasing order).

	**RSC-Fr before pandemic (*****N*** = **21,441)**			**RSC-Fr during pandemic (*****N*** = **21,405)**			**Change in RSC-Fr (*N* = 21,120)**
	***M* (SD)**	**Low (“0–4”) RSC-Fr, %**	**Highest (“10”) RSC-Fr, %**	***M* (SD)**	**Low (“0–4”) RSC-Fr, %**	**Highest (“10”) RSC-Fr, %**	***M* (SD)**
Germany	8.67 (2.1)	4.0	49.5	5 (3.1)	44.5	11.1	−3.4 (3.9)
Turkey	8.7 (2.0)	4.8	52.6	5.37 (3.1)	37.1	11.8	−3.3 (3.3)
Bangladesh	7.63 (3.1)	17.4	45.2	5.35 (3.5)	41.8	21.1	−2.3 (3.8)
Italy	8.73 (2.0)	4.6	54.5	6.56 (2.8)	20.9	19.6	−2.2 (3.1)
Albania	9.25 (1.5)	1.8	66.8	7.17 (2.7)	16.0	25.4	−2.1 (2.9)
Romania	8.97 (2.0)	4.9	63.9	7.4 (2.9)	16.5	32.7	−1.6 (3.1)
Chile	7.98 (2.8)	13.2	49.3	6.72 (3.3)	25.4	31.3	−1.3 (3.3)
Wales	8.43 (2.2)	6.8	48.0	7.20 (2.9)	18.4	30.6	−1.3 (2.8)
Taiwan	8.01 (2.1)	6.1	33.3	6.94 (2.7)	16.5	25.0	−1.1 (2.3)
Belgium	8.42 (2.5)	8.9	53.8	7.37 (2.9)	16.4	34.8	−1.1 (2.6)
Algeria	7.4 (3.3)	19.3	44.2	6.37 (3.6)	29.8	34.6	−1.0 (3.8)
Israel	8.35 (2.6)	10.8	54.2	7.37 (2.9)	19.2	37.2	−1.0 (3.1)
Russia	7.76 (2.9)	15.5	44.3	6.79 (3.3)	25.7	32.0	−1.0 (2.7)
South Korea	7.34 (1.5)	4.1	5.5	6.42 (1.9)	17.1	3.3	−0.9 (2.0)
Indonesia	8.31 (2.3)	7.7	43.9	7.66 (2.7)	13.6	34.9	−0.7 (2.5)
Estonia	8.68 (2.0)	5.3	51.1	8.10 (2.4)	10.1	40.3	−0.6 (2.1)
Finland	8.79 (1.9)	4.4	54.2	8.29 (2.2)	7.7	41.4	−0.5 (1.8)
Spain	8.15 (2.4)	7.7	43.4	7.82 (2.6)	12.9	37.1	−0.3 (2.8)
Total	8.3 (2.4)	8.3	47.4	6.99 (3.0)	20.3	28.7	−1.3 (3.0)

Country-specific analysis showed that RSC-Fr decreased in all countries. However, the starting point was different. Before the COVID-19 pandemic, the mean of RSC-Fr was very high (above 9) in Albania; in the majority of countries, it was at a high level, and only in South Korea, Algeria, Bangladesh, Russia, and Chile was it at the average level. However, during the pandemic, there remained no country with a very high level of RSC-Fr and there are only two countries—Finland and Estonia—with a high level of RSC-Fr.

The RSC-Fr decreased the least— < 1 point on the 0–10 scale—not only in Spain, Finland, Estonia, and Indonesia but also in South Korea and Russia. We called them countries with “almost no change.” During the COVID-19 pandemic, the RSC-Fr was at a high level only in Finland and Estonia, but they were already located at the top before the pandemic. In Spain and Indonesia, although being located in the middle before the pandemic, with almost no change, they were now at the top, despite having an average level of the RSC-Fr. South Korea and Russia had one of the lowest levels of RSC-Fr before, but with only little change, they were not among the lowest group of countries during the pandemic.

Children's RSC-Fr decreased slightly—1–2 points on a 0–10 scale—in Israel, Algeria, Belgium, Taiwan, Wales, Chile, and Romania. We called them countries with a “small decrease.” RSC-Fr decreased quite notably—2–3 points on a 0–10 scale—in Albania, Italy, and Bangladesh. We called them countries with a “notable decrease.” Before the COVID-19 pandemic, the highest RSC was in Albania, but, following a decrease, Albania was located in the middle. In Bangladesh, the RSC level remained one of the lowest before and during the pandemic.

Children's RSC-Fr decreased significantly—3–4 points on a 0–10 scale—in Turkey and Germany. We called them countries with a “major decrease.” In Turkey and Germany, the level of RSC was one of the highest before the COVID-19 pandemic, but the steepest decrease saw them located as the lowest during the pandemic.

As a robustness check, we compared the country means of two variables—“My friends are usually nice to me” and “Me and my friends get along well together”—based on data from the International Survey of Children's Well-Being 2018 and International Children's Worlds COVID-19 Supplement Survey 2021. It was possible to compare the means of Albania, Algeria, Belgium, Indonesia, Israel, Italy, Spain, and Wales. We found that, on average, the means declined 0.1–0.2 points on a 0–4 scale. In that group of countries, values declined most in Albania, corresponding to our results here.

### Profiles of change in children's subjective relational social cohesion

Next, we wanted to understand in more detail how children experienced the effects of the pandemic on their relationships with friends and family. Based on the change in RSC-Fr and RSC-Fa during the pandemic, we conducted a cluster analysis using country-pooled data. Five profiles of change in relational social cohesion emerged: (1) gainers in both RSC; (2) gainers in RSC-Fa and decliners in RSC-Fr; (3) no change in either RSC; (4) decliners in RSC-Fa and gainers in RSC-Fr; and (5) decliners in both RSC.

For the majority of the children (88.9%), both RSC-Fr and RSC-Fa did not change much during the pandemic (Table 5). By country, among different profiles of change, the proportion of “no changers” was most notable in Finland (97%), South Korea (95%), and Estonia (94%).

**Table 5:** Profiles (clusters) of change in children's relational social cohesion [average Silhouette = 0.6 (good)].

**Profiles by the change in RSC-Fr and RSC-Fa**	**Change in RSC-Fr**		**Change in RSC-Fa**		***N***	**%**
	**Mean**	**SD**	**Mean**	**SD**		
Gainers in both RSC	3.3	3.3	6.4	2.2	318	1.5
Gainers in RSC-Fa, decliners in RSC-Fr	−6.5	2.7	5.8	2.3	109	0.5
No change in either type of RSC	−1.0	2.4	−0.1	1.1	18,644	88.9
Decliners in RSC-Fa, gainers in RSC-Fr	6.4	2.8	−4.3	3.2	132	0.6
Decliners in both RSC	−5.9	3.0	−5.4	2.4	1,768	8.4
Total	−1.3	3.0	−0.5	2.2	20,971	100

The next most common cluster (8.4%) was labeled “decliners in both RSC” (both RSC types decreased during the pandemic). Among all the profiles, RSC-Fa decreased the most in the “decliners in both RSC” profile. By country, the proportion of “decliners in both RSC” was most notable in Germany (25%), Turkey (24%), and Bangladesh (18%).

The third most common cluster (1.5%) was “gainers in both RSC.” Among all the profiles of change, RSC-Fa increased the most in the “gainers in both RSC” profile. By country, the proportion of “gainers in both RSC” was most notable in Algeria (7%) and Spain (6%).

There were also small proportions of children whose RSC in the family increased but with friends decreased, and *vice versa*. Among all the change profiles, mean RSC-Fr decreased the most in the “gainers in RSC-Fa, decliners in RSC-Fr” profile and increased the most in the “decliners in RSC-Fa, gainers in RSC-Fr” profile.

### The quantity of children's relationships by profiles of change in relational social cohesion

In Table 6, we can see that the quantity of children's relationships differs by their profiles of change in relational social cohesion. “Gainers in both RSC” had to be at home all day because of COVID-19 significantly (*p* < 0.05) less likely and played or spent time outside more frequently than “decliners in both RSC.” However, “gainers in both RSC” were significantly more likely to have stated that they or somebody in their home got infected with COVID-19, and also had higher COVID-19 anxiety than “no changers.”

**Table 6:** Children's quantity of relationships by profiles of change in relational social cohesion (in all cases lower value indicates a lower quantity of relationships and *vice versa*).

		**Gainers in both RSC**	**Gainers in RSC-Fa, decliners in RSC-Fr**	**No-changers**	**Decliners in RSC-Fa, gainers in RSC-Fr**	**Decliners in both RSC**	**Kruskal–Wallis *H***	***N***
**Compulsory physical distancing from friends and a high density of contacts inside the family due to the confinement measures**								
… had to be in home all day because of COVID-19	Mean (SD)	1.71 (0.92)	1.54 (0.86)	1.59 (0.89)	1.52 (0.84)	1.50^2G, NC^ (0.85)	23.26***	20,457
	Median	1	1	1	1	1		
	yes %	60.7	70.4	68.4	70.9	72.9		
… could not attend school for many days	Mean (SD)	1.48 (0.81)	1.25^NC^ (0.63)	1.56 (0.88)	1.52 (0.84)	1.37^NC^ (0.76)	87.34***	20,615
	Median	1	1	1	1	1		
	yes %	72.0	84.9	70.2	70.2	79.9		
**In-person self-distancing from friends and a high density in the family due to infection or risk of infection**								
… me or somebody in my home got infected	Mean (SD)	2.47^NC^ (0.86)	2.53 (0.84)	2.64 (0.75)	2.54 (0.82)	2.58^NC^ (0.80)	26.43***	20,519
	Median	3	3	3	3	3		
	yes %	24.4	22.1	16.3	20.9	19.4		
… someone at home at high risk of getting very ill if gets infected	Mean (SD)	2.03 (0.96)	2.01 (0.96)	2.09 (0.95)	1.81^NC^ (0.93)	1.96^NC^ (0.96)	38.39***	20,349
	Median	2	2	3	1	2		
	yes %	43.9	45.5	41.2	54.3	47.8		
**In-person self-distancing from friends and a high density in the family due to COVID-19 anxiety**								
… COVID-19 anxiety	Mean (SD)	2.65^NC^ (1.07)	2.71 (1.08)	2.84 (1.00)	2.65 (1.13)	2.53^NC^ (1.08)	139.49***	19,905
	Median	2.86	3.00	3.00	2.71	2.71		
	< 2, %	24.6	22.1	19.1	30.8	29.4		
**Frequency of in-person or online interactions**								
… playing or hanging out outside	Mean (SD)	2.78 (1.80)	2.38 (1.89)	2.74 (1.72)	2.49 (1.80)	2.40^2G, NC^ (1.79)	65.05***	20,455
	Median	3	2	3	3	2		
	Once/ twice a week or less, %	44.8	55.1	45.6	49.2	54.6		
… meeting with friends online	Mean (SD)	2.46 (1.91)	2.34 (1.84)	2.38 (1.83)	2.43 (1.86)	2.17^NC^ (1.86)	21.42***	20,549
	Median	3	2	2	2	2		
	Once/ twice a week or less, %	49.0	54.2	53.1	52.8	58.6		
**New online friendships**								
… made new friends with other children online	Mean (SD)	1.38 (1.58)	1.58 (1.65)	1.33 (1.46)	1.74 (1.66)	1.21^NC, DG^ (1.46)	23.05***	20,439
	Median	1	1	1	1	0		
	Do not agree,%	48.4	43.8	44.6	39.0	50.1		

^2G^—significantly (*p* < 0.05; based on Mann-Whitney test) lower than gainers in both RSC; ^NC^—significantly lower than no changers, ^DG^—significantly lower than decliners in RSC-Fa and gainers in RSC-Fr.

***p < 0.001.

“Gainers in RSC-Fa, decliners in RSC-Fr” were significantly more likely to have been unable to attend school for many days than “no changers.” “Decliners in RSC-Fa, gainers in RSC-Fr” were more likely to agree than “decliners in both RSC,” that they made new friends with other children online but were significantly more likely to have someone at home at a high risk of getting very ill if they became infected than “no changers.”

Children who declined in both RSC were significantly more likely to have to be at home all day because of COVID-19 than children who gained in both RSC and children whose RSCs did not change during the pandemic. Moreover, “decliners in both RSC” were significantly more likely to not be able to attend school for many days, have family members or themselves be infected, have someone at home at high risk of getting very ill if they get infected, and had higher COVID-19 anxiety than “no changers.” “Decliners in both RSC” significantly less frequently played or hung out outside than “gainers in both RSC” and “no changers” and significantly less agreed that they made new friends with other children online during the pandemic compared to “no changers” and “decliners in RSC-Fa, gainers in RSC-Fr.”

### The quality of children's relationships by profiles of change in relational social cohesion

Considering the quality of children's relationships, in Table 7 we see that children's perceptions of safety, support, loneliness, boredom, autonomy, and “being listened to” differ by their profiles of change in relational social cohesion. “No changers” tend to have the most positive perceptions in all of these quality dimensions.

**Table 7:** Children's quality of relationships by profiles of change in relational social cohesion (in all cases lower value indicates a lower quality of relationships and *vice versa*).

		**Gainers in both RSC**	**Gainers in RSC-Fa, decliners in RSC-Fr**	**No-changers**	**Decliners in RSC-Fa, gainers in RSC-Fr**	**Decliners in both RSC**	**Kruskal–Wallis *H***	***N***
**Perceptions of safety**								
… feeling safe with my friends	Mean (SD)	2.50 (1.48)	2.30 (1.40)	2.64 (1.30)	2.44 (1.51)	2.41^NC^ (1.39)	48.55***	20,673
	Median	3	3	3	3	3		
	I do not agree or agree a little, %	27.7	32.7	21.3	27.6	28.9		
… feeling safe in home	Mean (SD)	3.13 (1.20)	2.89^NC^ (1.25)	3.31 (0.99)	2.90^NC^ (1.33)	3.20^NC^ (1.05)	42.83***	20,715
	Median	4	3	4	3	4		
	I do not agree or agree a little, %	12.9	15.9	7.0	17.3	9.1		
**Perceptions of support**								
… feeling support by some of my friends	Mean (SD)	2.23 (1.38)	1.85^NC^ (1.34)	2.26 (1.25)	2.02 (1.33)	1.99^2G, NC^ (1.30)	83.15***	20,338
	Median	2	2	2	2	2		
	I do not agree or agree a little, %	33.7	43.3	28.1	36.9	37.9		
… feeling support by some people I live with	Mean (SD)	3.00 (1.28)	3.00 (1.21)	3.19 (1.05)	2.62^NC^ (1.52)	2.98^NC^ (1.14)	77.95***	20,564
	Median	4	3	4	3	3		
	I do not agree or agree a little, %	15.7	11.5	8.6	27.0	12.0		
**Perceptions of loneliness**								
… feeling alone	Mean (SD)	2.80 (1.46)	2.35^2G, NC^ (1.53)	2.90 (1.33)	2.57 (1.51)	2.38^2G, NC^ (1.48)	228.93***	20,484
	Median	3	3	3	3	3		
	I agree a lot or totally, %	22.9	33.0	18.7	28.3	30.9		
**Perceptions of boredom**								
… feeling bored during the last two weeks	Mean (SD)	4.61 (3.94)	4.33 (3.81)	4.89 (3.38)	3.74^NC^ (3.55)	4.11^NC^ (3.48)	98.76***	20,281
	Median	5	5	5	3	4		
	0–4, %	49.5	49.0	46.5	60.2	55.8		
**Perceptions of autonomy**								
… satisfaction with the freedom you have	Mean (SD)	7.19^NC^ (3.28)	6.64^NC^ (3.53)	8.13 (2.35)	6.58^NC^ (3.49)	7.12^NC^ (2.92)	231.77***	20,252
	Median	8	8	9	8	8		
	Low (“0–4”) %	20.2	30.2	8.0	28.2	18.9		
**Perceptions of “Being listened to”**								
… my opinions about the COVID-19 are taken seriously in my home	Mean (SD)	2.14^NC^ (1.41)	1.93^NC^ (1.57)	2.38 (1.31)	1.96^NC^ (1.42)	2.26^NC^ (1.36)	37.22***	20,440
	Median	2	2	3	2	2		
	I do not agree or agree a little, %	33.0	47.5	26.3	41.0	30.9		

^2G^—significantly (*p* < 0.05; based on Mann-Whitney test) lower than gainers in both RSC; ^NC^—significantly lower than no changers.

***p < 0.001.

“Gainers in both RSC” were significantly more likely than “decliners in both RSC” to agree that they felt supported by some of their friends. In addition, they were less likely to agree than “gainers in RSC-Fa, decliners in RSC-Fr” and “decliners in both RSC” that they felt alone. However, “gainers in both RSC” were significantly less satisfied with the freedom they had and agreed less that their opinions about COVID-19 were taken seriously in their homes than “no changers.”

“Gainers in RSC-Fa, decliners in RSC-Fr” agreed significantly less that they felt safe at home, felt supported by some of their friends, that their opinions about COVID-19 were taken seriously in their home, and were less satisfied with the freedom they had than “no changers.” They also agreed more than “gainers in both RSC” and “no changers” that they felt alone.

“Decliners in RSC-Fa, gainers in RSC-Fr” agreed significantly less that they felt safe at home, felt supported by some people they live with, that their opinions about COVID-19 were taken seriously in their home, were less satisfied with the freedom they had, and felt more bored than “no changers.”

“Decliners in both RSC” agreed significantly less that they felt safe with their friends and at home, felt supported by some of their friends or by some people they live with, that their opinions about COVID-19 were taken seriously in their home, were less satisfied with the freedom they had, felt more bored, and agreed significantly more that they felt alone than “no changers.” They also agreed significantly less that they felt supported by some of their friends and agreed more that they felt alone than “gainers in both RSC.”

### Factors of the quantity and quality of children's relationships explaining their belonging to a gainers or decliners profile

To understand which of the relational factors help to explain belonging to a certain profile of RSC change, we performed multinomial logistic regression analysis. We outlined the quantity and quality of relationship factors that help to explain children's belonging to the “gainers” or “decliners” profile compared to “no changers.”

#### Gainers in both RSC

Mainly, the quantity-of-relationships factors help to explain children's belonging to a “gainers in both RSC” profile compared to “no changers” (Table 8). *Compulsory physical distancing from friends, and a high density of contacts inside the family due to the confinement measures* as relational factors help to explain children's belonging to the “gainers in both RSC” profile. More specifically, children who had to be at home all day were less likely “gainers in both RSC” compared to “no changers” than those who did not have to stay at home. Interestingly, children who were not sure if they “were not able to attend school for many days” were more likely “gainers in both RSC” compared to “no changers” than those who could attend school. In addition, *in-person self-distancing from friends* and *a high density in family due to infection or risk of infection* help to explain children's belonging to a “gainers in both RSC” profile. Children who were infected or if somebody in their home got infected with COVID-19 had twice higher odds than children with no infection experience to be a “gainer in both RSC” compared to “no changers.” *In-person self-distancing from friends* and *a high density in family due to COVID-19 anxiety* help to explain children's belonging to the “gainers in both RSC” profile. With a lower COVID-19 anxiety score, children were less likely “gainers in both RSC” compared to “no changers.” *The frequency of in-person or online interactions* and *making new friends online* did not help to explain children's belonging to the “gainers in both RSC” profile.

**Table 8:** Multinomial logistic regression model for predicting the likelihood (OR, odds ratio; SE, standard error) to be (1) gainer and (2) decliner in both RSC (*N* = 216 and 1,361, respectively) compared to no-changers (*N* = 15,052).

		**Gainers in both RSC**			**Decliners in both RSC**		
		***b***	**OR**	**SE**	***b***	**OR**	**SE**
Controls	Girls (ref: boys)	0.287*	1.333	0.140	0.012	1.013	0.058
	Access to the Internet	0.125	1.134	0.081	0.109***	1.115	0.033
	Not having own room (ref: having it)	0.136	1.145	0.151	0.024	1.024	0.064
Quantity: compulsory physical	Had to be in home all day (ref: had not to be)	−0.368*	0.692	0.155	0.112	1.118	0.070
distancing from friends and a high	Not sure if had to be in home all day (ref: did not have)	0.319	1.376	0.297	0.020	1.020	0.162
density of contacts inside the family due	Could not attend school for many days (ref: could attend)	0.352	1.422	0.182	0.417***	1.518	0.077
to the confinement measures	Not sure if can attend school for many days (ref: could attend)	0.864**	2.374	0.325	0.359*	1.431	0.172
Quantity: in-person self-distancing from	Me or somebody in my home got infected (ref: did not)	0.677***	1.969	0.164	0.223**	1.250	0.076
friends and a high density in the family due to infection or risk of infection	Not sure if I or somebody in my home got infected (ref: did not)	0.087	1.091	0.377	−0.113	0.893	0.162
	Had someone at home at high risk of getting very ill if got infected (ref: had not)	0.078	1.081	0.149	0.198**	1.219	0.063
	Not sure if had someone at home at high risk of getting very ill if got infected (ref: had not)	−0.468	0.626	0.299	0.114	1.121	0.112
Quantity: in-person self-distancing from friends and a high density in the family due to COVID-19 anxiety	COVID-19 anxiety	−0.157*	0.855	0.074	−0.204***	0.815	0.031
Quantity: frequency of in-person or online interactions	Playing or hanging out outside	0.042	1.043	0.042	−0.030	0.970	0.018
	Meeting with friends online	0.026	1.027	0.041	−0.012	0.988	0.018
Quantity: new online friendships	Made new friends with other children online	−0.079	0.924	0.052	−0.115***	0.891	0.022
Quality: perceptions of safety	Feeling safe with my friends	0.021	1.021	0.059	0.022	1.022	0.024
	Feeling safe at home	0.008	0.072	1.008	0.031	1.031	0.030
Quality: perceptions of support	Feeling support by some of my friends	0.046	1.047	0.063	−0.068**	0.934	0.026
	Feeling support by some people I live with	−0.006	0.994	0.071	−0.040	0.961	0.029
Quality: perceptions of loneliness	Feeling alone	0.039	1.040	0.055	−0.142***	0.868	0.022
Quality: perceptions of boredom	Feeling bored	−0.034	0.966	0.021	−0.025**	0.976	0.009
Quality: perceptions of autonomy	Satisfaction with the freedom you have	−0.104***	0.901	0.027	−0.084***	0.920	0.011
Quality: perceptions of “being listened to'	My opinions about the Coronavirus are taken seriously in my home	−0.081	0.922	0.056	−0.086***	0.918	0.024
Intercept		−3.521***		0.463	−0.819***		0.192
*N*		216			1,361		
Nagelkerke *R*^2^		0.067					

*p < 0.05.

**p < 0.01.

***p < 0.001.

Among different quality factors, only *autonomy perceptions* helped to explain children's belonging to the “gainers in both RSC” profile. With higher satisfaction with the freedom they have, children were less likely to be the “gainers in both RSC” compared to “no changers.”

Among controls, we found that girls were more likely than boys to be “gainers in both RSC” compared to “no changers.”

#### Decliners in both RSC

Almost all the quantity-of-relationship factors help to explain children's belonging to the “decliners in both RSC” profile compared to “no changers.” *Compulsory physical distancing from friends* and *a high density of contacts inside the family due to the confinement measures* are factors that help to explain children's belonging to the “decliners in both RSC” profile. More specifically, children who could not or were not sure if they were able to attend school for many days were more likely to be “decliners in both RSC” compared to “no changers” than children who could attend school. *In-person self-distancing from friends* and *a high density in family due to infection or risk of infection* are factors that help to explain children's belonging to the “decliners in both RSC” profile. Children who were infected or if somebody in their home got infected with COVID-19 had 1.3 times higher odds than children with no infection experience to be “decliners in both RSC” compared to “no changers.” Children who had someone at home at a high risk of getting very ill if infected were more likely to be “decliners in both RSC” compared to “no changers” than children who did not have such a family member. *In-person self-distancing from friends* and *a high density in family due to COVID-19 anxiety* help to explain children's belonging to the “decliners in both RSC” profile. With a lower COVID-19 anxiety score, children were less likely to be “decliners in both RSC” compared to “no changers.” *New online friendships* helped to explain children's belonging to the “decliners in both RSC” profile. Children who agreed that they made new friends with other children online were less likely to be “decliners in both RSC” compared to “no changers.” *The frequency of in-person or online interactions* did not help to explain children's belonging to the “decliners in both RSC” profile.

In addition, the quality-of-relationship factors helped to explain children's belonging to the “decliners in both RSC” profile compared to “no changers.” *Support perceptions* help to explain children's belonging to the “decliners in both RSC” profile. More specifically, children who agreed more that they feel supported by some of their friends were less likely to be “decliners in both RSC” compared to “no changers.” *Perceptions of* “*being listened to*” help to explain children's belonging to the “decliners in both RSC” profile. Children who agreed more that their opinions about COVID-19 were taken seriously in their homes were less likely to be “decliners in both RSC” compared to “no changers.” *Autonomy perceptions* help to explain children's belonging to the “decliners in both RSC” profile. With higher satisfaction with the freedom they had, children were less likely to be “decliners in both RSC” compared to “no changers.” *Perceptions of loneliness and boredom* help to explain children's belonging to the “decliners in both RSC” profile. Children who agreed less that they feel alone and who feel less bored were less likely to be “decliners in both RSC” compared to “no changers.” *Perceptions of safety* did not help to explain children's belonging to the “decliners in both RSC” profile.

Among controls, we found that children who had more frequent access to the Internet were more likely to be “decliners in both RSC” compared to “no changers.”

## Discussion and conclusions

The COVID-19 pandemic caused major changes in people's everyday routines. Both adults and children had to disconnect from in-person contact because of the confinement measures. Children had to cope with school closures, adapt to distance learning, and be separated from friends; many parents stayed out of work or worked remotely. Previous studies indicated that children's relational social cohesion in the family (e.g., Kutsar and Kurvet-Käosaar, 2021) and with friends (e.g., Choi et al., 2021; Stoecklin et al., 2021) may have decreased during the pandemic, but some children still described their positive experiences gained from the confinement measures of social distancing (Salin et al., 2020). Mostly, these studies are qualitative or focus on a single country and carry an exploratory character. In this study, we aimed to examine how the COVID-19 pandemic has affected children's subjective relational social cohesion (RSC) with family and friends, including the role of the quantity and quality of their relationships based on more than 20,000 primarily 9–13-year-old children's data from 18 countries.

Our analyses confirmed the decrease in familial and external relational social cohesion (measured as satisfaction with relationships with friends before and during the pandemic). In all the sample countries, children's satisfaction with relationships with friends and family members with whom they live together changed: low assessments (0–4 points on a 10-point scale) increased while the highest assessments (10 points on the same scale) decreased. The decrease was most notable in Germany, Turkey, and Bangladesh, which require further in-depth analysis. However, it is interesting that, in these countries, children reported most often that they could not attend school for many days while in Finland and Estonia, where the decrease was one of the smallest, it was the opposite. Even when the change was of different sizes in different countries, we conclude that the relational social cohesion of children was at risk during the pandemic.

Compared to relational cohesion with friends, the decrease was noticeably smaller inside the family, confirming our hypothesis. Former qualitative studies can help to explain this difference. For example, Kutsar and Kurvet-Käosaar (2021) and Stoecklin et al. (2021) describe the pros and cons of the pandemic situation from children's perspectives. On the one hand, families had new time reserves to develop their quality time and consolidate. They started with new joint activities, such as playing games together and cooking. This evidence refers to new resources to bolster familial relational social cohesion. Still, the lasting density at home and the diverse multiple tasks that family members performed separately before the pandemic in different life domains were suddenly concentrated in the same space—the family. On the other hand, the density of interactions and time spent together started to endanger the quality of mutual relationships, e.g., it resulted in increasing conflicts and even violence (e.g., Biroli et al., 2020; Lebow, 2020; Lee et al., 2020; Kutsar and Kurvet-Käosaar, 2021; Magson et al., 2021). Moreover, family members in their mutual conversations started to blow up COVID-19 anxiety and perceived the necessity of self-distancing from fragile elderly members of the extended families (Kutsar and Kurvet-Käosaar, 2021). Living in close proximity put children at risk of losing the personal freedom and autonomy they were used to before the pandemic outbreak. The latter is a risk factor against positive family consolidation, described also, for example, in the study of Stoecklin et al. (2021), according to whom the sources of these tensions sprang from the lack of children's own space and privacy and unusual parental control as an impediment to their autonomy. In sum, there were positive and negative challenges to changing intra-family relational social cohesion; however, positive aspects neutralized some negative effects of living densely together (e.g., Mariani et al., 2020). This explains why the decrease in familial RSC was not very high.

The pandemic restrictions and especially the confinement measures disconnected people of different ages from social life and endangered their external relational social cohesion. Previous studies showed that children and youth have a strong orientation to developing their social relationships, and compulsory disconnections from friends during the pandemic became their major concern (Meuwese et al., 2017; Ellis et al., 2020; Choi et al., 2021; Kutsar and Kurvet-Käosaar, 2021; Magson et al., 2021; Stoecklin et al., 2021). This was the main reason for dreaming about going back to school as a solution (e.g., Kutsar and Kurvet-Käosaar, 2021; Stoecklin et al., 2021). Our analyses confirmed the bigger decrease in external relational social cohesion (measured as satisfaction with relationships with friends before and during the pandemic) compared to changes in family cohesion. For example, in Germany, where children perceived the biggest decline in satisfaction with relationships with friends, 49.5% of children were totally satisfied with their friends retrospectively, but after a year of living with the pandemic, only 11.1% were totally satisfied; meanwhile, the group with low satisfaction increased during the pandemic from 4.0 to 44.5%. Still, children in Germany also went through a noticeable negative change in satisfaction with the relationships with their family members: the group with low satisfaction increased from 3% before the pandemic to 10.9% a year later; the totally satisfied group changed from 51% prior the pandemic to 33.8% during it. In sum, new circumstances reshaped the social relationships of children and disrupted their spontaneous embeddedness into adults” and peer networks, their active “knitting” of the “orb web,” as Corsaro (1997) calls it.

Immediately after the onset of the pandemic and the implementation of confinement measures, several studies (e.g., Loades et al., 2020) started to document emerging mental health problems in children as a pandemic outcome. Younger children missed their friends from school, older youth felt bored and longed for romantic relationships; young people of different ages felt lonely and complained about the loss of autonomy and freedom (e.g., Stoecklin et al., 2021). In the present analyses, we were interested in the clusters of children who shared similar assessments about their confinement experiences. The cluster analysis revealed five clusters of children which we consider as their profiles of change in relational social cohesion. Most children (88.9%) belonged to the “no changers” profile, as they did not report notable changes in their relational cohesion appraisals (in Finland even 97%, 95% in South Korea, and 94% in Estonia). At first glance, the high percentage belonging to this “no changers” profile was surprising to us, especially when thinking of the patterns of evidence revealed in qualitative studies with children. Still, the homeostatic principle described by Cummins (2014) can explain it: subjective wellbeing seems to be stable unless there are lasting negative events affecting children's lives, especially when the closest family is concerned. Our analyses also demonstrated smaller changes in relational social cohesion in the family compared to external relational cohesion (satisfaction with relationships with friends). Interestingly, “no changers” tend to have more positive perceptions in all the quality-of-relationships factors, especially in the case of perceptions of autonomy and “being listened to” compared to children belonging to other profiles.

The second most common profile of change was “decliners in both RSC”—for 8.4% of children, relational social cohesion with family and friends decreased during the pandemic. Almost all the selected quantity and quality of relationships factors help to explain children's belonging to a “decliners in both RSC” profile compared to “no changers.” However, our analyses revealed that, for 1.5% of children, relational social cohesion with family and friends increased during the pandemic. These children belong to the “gainers in both RSC” profile. Mainly the quantity of relationships factors, and among different quality of relationship factors, only perceptions of autonomy (“satisfaction with freedom'), help to explain children's belonging to the “gainers in both RSC” profile compared to “no changers.” Being positive about the freedom they had during the pandemic seems to have helped children to follow their preferences of social interactions, especially at times of school closures during the pandemic. There are children whose “normalcy” was withdrawn (e.g., those on the autism spectrum—see Locke et al., 2010; Kasari et al., 2011) or those who do not like going to school because of bullying or learning problems (see, e.g., Hall et al., 2021). During the pandemic, they could experience social distance from classmates and teachers as a personal freedom. According to former studies, they diverted from an adultist normative approach—from children who are embedded in intra- and extra-familial networks and “weave their webs” as active social actors. Moreover, being relationally socially coherent, they develop peer cultures, a sense of belonging, trust in other people, social skills, and influence social change (Corsaro, 1997). We suppose that children who enjoyed more freedom during the pandemic may have problems with returning to school at the end of the social-distancing measure. This aspect should be taken into consideration as a risk factor, especially in the case of children with special educational needs and children who, before the pandemic, experienced neglect or being withdrawn, i.e., those with low relational social cohesion (see, e.g., Locke et al., 2010; Kasari et al., 2011; Hall et al., 2021).

Compulsory measures and/or self-distancing due to infection/risk of infection and “COVID-19 anxiety” seemed to be important factors explaining the different experiences of “gainers” and “no changers,” on the one hand, and “decliners” on the other hand. Children who declined most in RSC during the pandemic had to be at home all day because of COVID-19 significantly more than “gainers” and “no changers,” including significantly less frequently playing or spending time outside due to the restrictions. Moreover, “decliners in both RSC” were significantly more likely to not be able to attend school for many days, have someone at home at a high risk of getting very ill if they got infected, and have higher COVID-19 anxiety than “no changers.” Those who had to stay or decided to stay at home declined in RSC because of the lack of in-person contacts outside the home. With reference to Corsaro's (1997) approach, the social-distancing measures severely disturbed children's customary way of life (their subjective normalcy) and development, i.e., their active embeddedness into social networks.

However, children whose social distancing was less strict were more likely to develop their relational social cohesion even during the pandemic: they have a higher probability of belonging to the group of “gainers in both RSC” compared to the “no changers.” Compulsory social distancing from friends and the risks of decreasing mental health are related to each other. Several studies were carried out about the importance of friends in children's social lives and personal development (e.g., Sakyi et al., 2014; Schwartz-Mette et al., 2020 for a review). Pandemic social-distancing confinement measures put active in-person friendships on hold (e.g., Stoecklin et al., 2021), thus also endangering the relational social cohesion of children beyond the family framework.

Stoecklin et al. (2021), in their study, refer to children's strategies of compensating for the lack of direct contact with friends with contact using IT devices; some children were even able to make new online friends to reshape their networking routines. We found that “decliners in both RSC” were less active in making new friends online (in contrast to the trend of increasing virtual contacts to compensate for the missing in-person communication, e.g., Ellis et al., 2020; Munasinghe et al., 2020; Sañudo et al., 2020). Our analysis showed that access to the Internet does not always mean maintaining contact with peers: children who had more frequent access to the Internet were more likely to be “decliners in both RSC” compared to “no changers.” Again, previous qualitative studies (Kutsar and Kurvet-Käosaar, 2021; Stoecklin et al., 2021) can help to explain this. In previous studies, children have admitted that they spend long hours on the computer surfing or playing games alone; thus, they were diverted from their own former normalcy. The latter cannot promote either socializing with peers or doing things together with family members. Instead of maintaining relational social cohesion or compensating it by making new friends online, these children choose self-distancing. According to children, “friendships from distance” cannot compensate for real in-person communication (Stoecklin et al., 2021; Larivière-Bastien et al., 2022).

Vogel et al. (2021) documented the decrease in social support from peers shortly after the lockdown. Due to the disconnection from friends, “decliners in both RSC” also felt less support. This evidence also agrees with the findings of Jiao et al. (2020), Okruszek et al. (2020), Singh and Singh (2020), and others. Moreover, several studies (e.g., Loades et al., 2020; Kutsar and Kurvet-Käosaar, 2021) stressed that social-distancing measures negatively impacted children's friendships and caused sadness and feelings of loneliness and boredom. Our study confirmed these results, as children who agreed more that they feel alone and bored were more likely “decliners in both RSC” compared to “no changers.” For “decliners in both RSC,” relational social cohesion in the family also decreased due to perceptions of having less freedom and autonomy, and that their opinions about COVID-19 were taken seriously in their home. We considered whether the “decliners in both RSC” had to divert from their pre-pandemic normalcy the most. However, this needs more in-depth research.

The cluster analysis also revealed small groups of children who gained higher familial cohesion and experienced loss in connections with friends (0.5%) or, conversely, gained higher extra-familial connections and felt loss in intra-familial ones (0.6%). Interestingly, those who gained closeness in the family lost most of their satisfaction with friends, and vice versa. It seems that these findings uncover some compensatory mechanisms that need further exploration.

There are several limitations to our study. The International Children's Worlds COVID-19 Supplement Survey took place during the pandemic when confinement measures shaped children's lives. During the data collection, many children stayed at home because of school closures. On the one hand, we documented their acute perceptions about the pandemic, but the relevance of the retrospective appraisals of relationships before the pandemic can be debated. Unfortunately, we do not have so-called baseline data (the same respondents answering similar questions before the pandemic). However, we do not regard this as a serious limitation because, in our opinion, following the interpretative essence of subjective wellbeing, people act according to their perceived reality, not objective circumstances, and, consequently, should be trusted. Moreover, a quantitative approach allows the exploration of social phenomena, to a certain extent. As we started with reference to several qualitative studies about the children's experiences of the pandemic, following the present discussion, we concluded that the subjective normalcy of children can differ from adults” normative understandings of relational social cohesion and, thus, should be further studied in-depth. Second, although our analyses are based on a unique and novel multinational database with wide geographical coverage, most countries did not have representative samples, and data-collection methods varied between countries. Moreover, the sample sizes vary considerably, from 590 in Germany to 2,422 in Belgium. This must be considered when interpreting the results of country-pooled analyses. Third, we had too few children belonging to “gainers in RSC-Fa, decliners in RSC-Fr” and “decliners in RSC-Fa, gainers in RSC-Fr” profiles to explore in further detail what relational factors help to explain their belonging to these profiles. Further in-depth contextual analyses would be helpful. Fourth, due to the small *N* in profiles other than “no changers,” it was not possible to explore the role of relational factors in belonging to a certain RSC change profile in each individual country.

To conclude, our analyses revealed that children experienced social-distancing measures during the pandemic differently. Almost one-tenth of children, as an average across the sample countries, have perceived significant loss in relational social cohesion. In some countries, such as Germany, Turkey, and Bangladesh, this percentage reaches one-fourth of children whose mental health should be the careful focus of psychologists, mental health practitioners, and other aid professionals. Our study confirmed the importance of keeping schools open not only with the aim of better educational outcomes but especially in terms of protecting relational social cohesion and the mental wellbeing of children. This evidence is echoed among policymakers in Estonia. Any future closure of schools should be avoided to prevent an extreme emergency because the negative outcomes of school closures and the social distancing of the whole population outweigh its positive aspects.

## Data availability statement

First version of Children's Worlds COVID-19 Supplement survey dataset was released in Spring 2022 and at moment it is available only for members of the countries' survey teams.

## Ethics statement

Active informed consent was obtained from all subjects involved in the study. Written informed consent to participate in this study was provided by the participants' legal guardian/next of kin.

## Author contributions

ON had a lead role in data analysis. All authors contributed equally to the drafting of the article.

## Funding

The research carried out for this publication was supported by a grant from the Estonian Research Council (Grant No. PRG700).

## Conflict of interest

The authors declare that the research was conducted in the absence of any commercial or financial relationships that could be construed as a potential conflict of interest.

## Publisher's note

All claims expressed in this article are solely those of the authors and do not necessarily represent those of their affiliated organizations, or those of the publisher, the editors and the reviewers. Any product that may be evaluated in this article, or claim that may be made by its manufacturer, is not guaranteed or endorsed by the publisher.
